# The critically-ill pediatric hemato-oncology patient: epidemiology, management, and strategy of transfer to the pediatric intensive care unit

**DOI:** 10.1186/2110-5820-2-14

**Published:** 2012-06-12

**Authors:** Pierre Demaret, Geraldine Pettersen, Philippe Hubert, Pierre Teira, Guillaume Emeriaud

**Affiliations:** 1 Division of pediatric critical care medicine, Department of Pediatrics, Sainte-Justine Hospital, Chemin de la Côte-Sainte-Catherine, Montreal, H2J3V6, Canada; 2 Division of pediatric critical care medicine, Hôpital Necker-Enfants Malades, Rue de Sèvres, 75007, Paris, France; 3 Division of pediatric hemato-oncology, Department of Pediatrics, Sainte-Justine Hospital, Chemin de la Côte-Sainte-Catherine, Montreal, H2J3V6, Canada

**Keywords:** Oncology, Hematology, Cancer, Stem cell, Transplantation, Graft, Child, Pediatrics, Critical care, Intensive care

## Abstract

Cancer is a leading cause of death in children. In the past decades, there has been a marked increase in overall survival of children with cancer. However, children whose treatment includes hematopoietic stem cell transplantation still represent a subpopulation with a higher risk of mortality. These improvements in mortality are accompanied by an increase in complications, such as respiratory and cardiovascular insufficiencies as well as neurological problems that may require an admission to the pediatric intensive care unit where most supportive therapies can be provided. It has been shown that ventilatory and cardiovascular support along with renal replacement therapy can benefit pediatric hemato-oncology patients if promptly established. Even if admissions of these patients are not considered futile anymore, they still raise sensitive questions, including ethical issues. To support the discussion and potentially facilitate the decision-making process, we propose an algorithm that takes into account the reason for admission (surgical versus medical) and the hemato-oncological prognosis. The algorithm then leads to different types of admission: full-support admission, “pediatric intensive care unit trial” admission, intensive care with adapted level of support, and palliative intensive care. Throughout the process, maintaining a dialogue between the treating physicians, the paramedical staff, the child, and his parents is of paramount importance to optimize the care of these children with complex disease and evolving medical status.

## Review

Children represent only 1% of all patients with malignant neoplasms [1]. Nevertheless, in developed countries, cancer is the second most common cause of death in children older than age 1 year, after trauma [2]. Tumor-related issues as well as the intensity of therapy itself can lead to severe and life-threatening complications that may require admission to a pediatric intensive care unit (PICU).

Historically, these children have been regarded as poor candidates for intensive care. Given their grim prognosis [3,4], their admission to the PICU raised difficult issues, ethically and operationally. During the past decades, there has been a marked improvement in the prognosis of children with cancer (5-year survival increasing from approximately 40% in the 1970s to approximately 80% in the 2000s [1,5]) as well as their intensive care outcome [6-8].

The objectives of this review are to characterize the specificities of the critically ill child with cancer, to review the organ support strategies that can be offered, and to assist pediatricians, hemato-oncologists, and pediatric intensivists in transitioning these patients to the PICU. Given their specific challenges, children who undergo hematopoietic stem cell transplantation (HSCT) will be discussed separately.

### **The pediatric hemato-oncologic patient (non-HSCT) admitted to the PICU**

#### **Epidemiology**

One of every three or four children with cancer is admitted to the PICU at least once during the course of their illness [9,10]. Children with cancer account for less than 10% of all PICU admissions [11-15].

### **Reasons for admission**

The main reasons for admission to the PICU are listed in Table 1. Sepsis and respiratory failure are responsible for approximately two-thirds of nonsurgical PICU admissions [12,15-17].

**Table 1:** Reasons for admission to the pediatric intensive care unit (PICU)

	**Pediatric hemato-oncology patients without HSCT**	**Children post- HSCT**
Postoperative care (% of PICU admission)	72% [11]	4–16% [18,19]
Medical reason^a^		
Respiratory failure	26–58% [10,15,17,20]	33–88% [19,21-23]
Airway compression^b^ (% of respiratory failure)	0–48% [10,17,20]	0% [19,21-23]
Lung disease (% of respiratory failure)	52–100%^c^[10,15,17,20]	100%^d^[19,21-23]
Severe sepsis/septic shock	8–36% [10,15,17,20,24]	21–36% [18,19,21,23]
Neurological problem	10–31% [10,15,17,20,24]	3–20% [18,19,21,25]
Renal dysfunction	5–15% [10,15,20]	5–8% [21,22,25]
Tumor lysis syndrome	5–8% [10,15]	

*HSCT* = hematopoietic stem cell transplantation.

^a^Percentages indicate the proportion of medical admissions only; ^b^airway compression by a tumor or by a mediastinal mass; ^c^mainly due to infection; ^d^possible etiologies are infectious pneumonia, idiopathic pneumonia, bronchiolitis obliterans, pulmonary hemorrhage, pulmonary edema, or GVHD.

### **Mortality and prognostic factors**

Mortality is primarily influenced by the type of admission (surgical vs. nonsurgical). Children admitted postoperatively have a very low mortality rate (0-4%) [10,11,17]. Because their prognosis does not differ from that of the general population of intensive care, they are excluded from the majority of the populations studied.

Mortality of medical cases is higher. The large multicenter study by Dalton et al. (n = 802) demonstrated a 13.3% mortality rate for children with cancer admitted for nonsurgical causes (30/226) [11]. Smaller single-center pediatric series, considering patients admitted to the PICU in the 2000s, have reported a mortality rate ranging from 15% to 20% [15,24]. These results, although higher than the mortality rate of the general PICU population, are encouraging compared with the 50% mortality rate reported in the 1980s [16].

As with the general PICU population, the degree of multiorgan failure is systematically related to prognosis; mortality exceeds 70% if three or more organs are involved [10,17,20,24]. The use of mechanical ventilation and/or inotropic support, related to respiratory and/or cardiovascular insufficiency, are other important prognostic factors [10,15,20,24]. Their combination is associated with a worse prognosis, with mortality reaching 54% to 100% [11,17,20]. It should be noted that children ventilated because of airway compression have a better outcome than those ventilated for lung disease [10,12,20].

Children admitted to the PICU at the time of diagnosis of oncologic disease, before initiation of chemotherapy, seem to have a better prognosis than those admitted later (8% vs. 34%, *p* = 0.06) [10]. This difference may be due to differences in the reasons for admission between these two groups and to the toxicity of chemotherapy itself.

Some authors also have reported that the type of cancer influences PICU mortality: children with solid tumors have a lower mortality rate than those with hematological malignancies [15,24].

### **Cancer and respiratory failure in the PICU**

Respiratory failure is a major cause of PICU admission for children with cancer (Table 1). The term “respiratory failure” is not always clearly defined in the literature addressing this issue. Acute respiratory distress syndrome (ARDS) should be defined according to the *American-European Consensus Conference on ARDS criteria*[26]: acute onset, PaO_2_/FiO_2_ ≤ 200 mmHg (regardless of positive end-expiratory pressure level), bilateral infiltrates on chest radiograph, and no evidence of left atrial hypertension. The acute lung injury (ALI) criteria only differ by the level of PaO_2_/FiO_2_, which has to be ≤ 300 mmHg. The SpO_2_/FiO_2_ ratio could be used in the evaluation of lung disease severity as a surrogate of the PaO_2_/FiO_2_ ratio if an arterial catheter is not available [27].

Noninvasive ventilation (NIV) is of particular interest in these patients who are highly susceptible to infections, because it does not breach the respiratory barrier. The benefits of NIV for immunocompromised patients have been documented in both adults [28-31] and children [32,33].

In a retrospective study of 239 children with cancer admitted to the PICU for respiratory failure, Pancera et al. [32] compared children ventilated for at least 24 hours with NIV (nasal biphasic positive airway pressure) (n = 120) with children ventilated invasively (without prior NIV or with prior NIV < 24 hours) (n = 119). The success rate of NIV, defined as the absence of subsequent endotracheal intubation, was 74%. In the multivariate analysis, predictors of failure of NIV were: cardiovascular dysfunction, therapeutic intervention score (TISS) ≥ 40, and presence of a solid tumor. Forty-six patients (39%) in the invasively ventilated group and 93 (77%) in the NIV group survived to PICU discharge (*p* < 0.001). There was a significant difference in the 30-day survival between the invasively ventilated group (23.3%) and the NIV group (47%; *p* < 0.0001). It is important to note that these two groups were derived from standard practice rather than randomization. Therefore, the results need to be interpreted with caution.

Two other retrospective studies by Schiller et al. [33] and Piastra et al. [34] reported data from 14 pediatric hemato-oncology patients with ALI and 23 pediatric hemato-oncology patients with ARDS, respectively. All of these patients received NIV via a full-face mask or a helmet. Intubation was avoided in 12 of 14 and 13 of 23 patients, respectively. Ten of the 12 intubated patients in the two studies died eventually compared with 1 of the 25 nonintubated patients.

Whereas a few randomized, controlled trials conducted in the adult population have shown significant benefits of early implementation of NIV in immunocompromised patients [28,30,31], no randomized trials have been conducted in children with cancer. Studies previously discussed have a significant potential for selection biases (the children who received NIV could be less severely ill than those who did not) and for information biases, inherent to their retrospective nature. However, considering potential complications related to invasive ventilation in the hemato-oncology children, and in light of the benefits of early NIV documented in adults, we believe it is important to consider a trial of NIV in this category of patients.

Despite the benefits of NIV, a difficult question remains: when is the appropriate time to declare NIV a failure and to initiate invasive ventilation in a pediatric hemato-oncology patient whose respiratory function is not improving? Invasive ventilation is documented as a poor prognostic factor among these children but delaying intubation also could worsen their course. To balance these effects, close monitoring is essential in all cases, and a possible switch to invasive ventilation should be discussed if there is no improvement after the first 2 hours of ventilation [35]. The criteria identified as predictive of NIV failure in the general PICU population are the presence of a second organ failure [36], pH < 7.25 after 2 hours of treatment [37], the need for a high level of support (mean pressure > 12 cmH_2_O or FiO_2_ > 0.6) [38], and the presence of ARDS [39].

### **Cancer and sepsis**

Several factors predispose the child with cancer to sepsis [40,41]: the chemotherapy regimens responsible for prolonged periods of marrow aplasia and disruption of skin and mucosal barriers, the type of tumor (hematological malignancies having a greater risk than solid tumors), the stage of the disease, neutropenia, as well as the presence of comorbidities and indwelling catheters.

Children with cancer in septic shock seem to have a prognosis similar to that of children without cancer, according to work by Pound et al. [40]. In this study, 69 pediatric oncology patients admitted to the PICU with a diagnosis of septic shock (based on the presence of hypo- or hyperthermia, tachycardia, tachypnea and hypotension, and evidence of perfusion abnormalities or organ dysfunction after adequate fluid resuscitation) were compared with a group of age- and gender-matched nononcology patients admitted to the PICU with septic shock during the same time period. No significant difference could be demonstrated with respect to survival status at PICU discharge (16% vs. 12% respectively, *p* = 0.67) nor at 30 days post-PICU discharge (23% vs. 15%, *p* = 0.38). However, there was a significant difference in mortality at 6 months post-PICU discharge between the two groups (43% vs. 16%, *p* = 0.01), explained by the underlying disease in the oncology patients, with all deaths being secondary to disease progression.

Similar results were found by Kutko et al. [42] who reviewed data from 96 episodes of septic shock (defined as hypo- or hyperthermia with signs of altered perfusion and/or hypotension) in 80 PICU patients. Although this study was not adequately powered for such a comparison, there was no difference in mortality rates between children with cancer (n = 68) and without (n = 28; 12% vs. 18%, respectively; *p* = 0.43).

The definitions of sepsis, severe sepsis, and septic shock in children were established in 2005 by the *International Pediatric Sepsis Consensus Conference*[43]. Systemic inflammatory response syndrome (SIRS) is defined by the presence of at least two of the followings: hypo- or hyperthermia, tachycardia or bradycardia, tachypnea or mechanical ventilation, and elevated or depressed leukocyte count. *Sepsis* is defined by SIRS in the presence of suspected or proven infection. *Sepsis* is qualified as *severe* in case of cardiovascular dysfunction, acute respiratory distress syndrome, or two or more other organ dysfunctions, whereas *septic shock* is defined by the association of sepsis and cardiovascular dysfunction. Because recruitment of patients in both studies evaluating children with cancer and septic shock preceded the consensus conference [40,42], the authors based the septic shock criteria on the Task Force on Hemodynamic Support from the American College of Critical Care Medicine [44]. These former criteria correspond to the consensus conference definitions of severe sepsis or septic shock, allowing comparison with patients admitted to our PICU with such diagnoses.

The management of children with cancer in septic shock does not differ substantially from that of other children and should follow the recommendations of the Surviving Sepsis Campaign [45]. Broad-spectrum empirical antibiotic therapy must be started immediately. Administration of intravenous immunoglobulin may be considered in children with severe sepsis (weak recommendation), although a recent meta-analysis does not recommend their use in current practice in the adult population [46]. According to international guidelines [47,48], hematopoietic growth factors should be part of the treatment of febrile neutropenia in patients at high risk of infectious complications (expected prolonged (>10 days) or profound (<0.1 × 10^9^/L) neutropenia, uncontrolled primary disease, pneumonia, hypotension, and multiorgan dysfunction) [47] or in patients unresponsive to antibiotics or with life-threatening complications [48]. Granulocyte transfusions are not recommended as a routine treatment and should be reserved for special situations [49].

Finally, extracorporeal blood purification therapies, such as high-volume hemofiltration, hemoadsorption with sorbents, such as polymyxin B, or plasmapheresis have been proposed for adults with sepsis given their immunomodulation potential associated with improvement of different physiologic parameters [50,51]. However, important questions regarding these strategies remain unanswered, and evidence of their usefulness in pediatrics is absent. Large trials are required before extracorporeal blood purification can be considered for standard clinical practice [50,51].

### **Cancer and extracorporeal life support (ECLS)**

Previously, many have argued that patients with cancer should not receive ECLS [52]. Recently, Gow et al. examined the Extracorporeal Life Support Organisation data from 1992 to 2007 pertaining to patients with a diagnosis of cancer and younger than age 21 years at the time of ECLS (n = 107) [53]. Table 2 displays the mortality rates of these patients.

**Table 2:** **Mortality of children with cancer placed on ECMO and comparison with patients without cancer (from**[53]**)**

	**Cancer patients**	**Noncancer patients**	***p*value**
All ECMO			
ECMO mortality	58% (62/107)		
Hospital mortality	65% (70/107)		
Pulmonary ECMO			
ECMO mortality	58% (50/86)	35%	<0.0001
Hospital mortality	64% (55/86)	44%	<0.0002
Cardiac ECMO			
ECMO mortality	57% (8/14)	39%	0.17
Hospital mortality	71% (10/14)	55%	0.22

*ECMO* = extracorporeal membrane oxygenation.

^a^Includes 86 pulmonary ECMO, 14 cardiac ECMO, and 7 ECMO for cardiopulmonary resuscitation.

This study showed that the rate of infectious complications was not higher than that observed in children without oncologic disease on ECLS (26% vs. 13–30%, respectively [54,55]). However, cardiovascular and renal complications (renal failure requiring replacement therapy, arterial hypertension, cardiovascular failure requiring inotropic support, and cardiorespiratory arrest) seem to be more common in children with cancer. Based on these data, it does not seem unreasonable to consider ECLS in children with cancer who meet the necessary criteria, including reversible pulmonary and/or heart failure persisting despite maximal medical therapy [53]. In practice, most centers do not exclude children with cancer from their ECLS programs as evidenced by a survey conducted in 118 centers belonging to the Extracorporeal Life Support Organisation. Seventy-eight percent of the surveyed centers stated that cancer was not a contraindication for ECLS, although it was considered a relative or absolute contraindication in 17% and 5% of the centers, respectively [53]. Despite the inherent selection bias (all respondents practiced ECLS), this survey demonstrates that ECLS may be considered as part of the PICU treatment offered to children with cancer.

### **Children with HSCT in the PICU**

Since the first description of a bone marrow transplant in a patient with leukemia in 1950 [56], indications and modalities of this therapy have considerably evolved and HSCT is now used for a wide array of malignant and nonmalignant diseases. Despite many advances, HSCT is still associated with a variety of complications that pose serious threats to transplant recipients (graft versus host disease (GVHD), severe sepsis, or organ dysfunction) [21,57].

### **Epidemiology**

In 2008, approximately 2,400 children benefited from HSCT in North America, with nearly 90% of transplants related to cancer [58,59]. The proportion of transplanted children admitted to the PICU varies between 10% and 20% [18,19,21-23,60,61] but a rate as high as 44% has been reported [6]. Patients who received an allogeneic transplant may be at greater risk of being admitted to the PICU than those who received an autologous transplant [21,22] although this is not a constant finding [8]. Patients transplanted late in the course of their disease may be at greater risk of requiring intensive care than those transplanted early [21,22]. Other risk factors for PICU admission identified in allogenic HSCT patients are the presence of GVHD and its severity [21,22], fluid overload [8], and engraftment syndrome [22].

### **Reasons for admission**

The main reasons for admission are listed in Table 1. Respiratory failure is the leading cause of admission [19,21-23] followed by severe sepsis/septic shock [18,19,21,23].

### **Mortality and prognostic factors**

The prognosis of children post-HSCT admitted to the PICU was previously very grim, especially when invasive ventilation was required. As shown in Table 3, the prognosis for these children has improved during the past decade; the PICU survival of ventilated children post-HSCT increased from <20% to >50% [6-8,18,19,21-23,25,60,61]. Survival at 6 months also seems to have improved, although data are scarce for children transplanted after 2000.

**Table 3:** Survival of children post-HSCT admitted to the pediatric intensive care unit (PICU)

**Author**	**n**		**Period of transplant**	**PICU survival**		**Survival ≥ 6 months after PICU discharge**	
	**Patients**	**Admissions**		**All**	**Ventilated**	**All**	**Ventilated**
Nichols [62]	39	39	1978–1988	44%	9%	NA	NA
Keenan [63]^a^	121	121	1984–1996	-	16%	-	7%
Hayes [61]	39	44	1987–1997	27%	15%	20.5%	12%
Schneider [60]	28	28	1989–1998	50%	36%	21%	14%
Jacobe [18]^b^	40	57	1994–1998	56%	42%	27%	13%
Hagen [64]^b^	86	98	1990–1999	-	41%	-	20%
Lamas [25]	44	49	1991–2000	37%	23%	13.6%	NA
Diaz [22]	42	42	1993–2001	31%	21%	17%	NA
Leung Cheuk [19]^b^	19	24	1992 V2002	54%	15%	16%	NA
Tomaske [23]	23	26	1998–2001	42%	15%	26%	NA
Gonzalez-Vicent [21]	36	36	1998–2002	47%	ND	44%	NA
Kache [6]							
All	81	NA	1992–2004	NA	NA	NA	NA
1992–1999	48	NA	1992–1999	NA	NA	6%	NA
2000–2004	33	NA	2000–2004	64%	59%	NA	NA
Van Gestel [7]*	35	38	1999–2007	-	58%	-	51%
Benoît [8]	19	19	2002–2004	68%	50%	NA	NA

*NA* = not available.

^a^Exclusion of patients ventilated < 24 h; ^b^includes postoperative patients (Jacobe: 2; Hagen: 5; Leung Cheuk: 4; Van Gestel: 1).

However, this improvement is questioned by some authors. In a retrospective analysis of a large American database comparing children transplanted in 1997, 2000, and 2003 (n = 5,699), Bratton et al. did not find any difference in mortality across the three study periods amongst children post-HSCT who required invasive ventilation (overall mortality 36%) [65]. In addition, a meta-analysis conducted by Van Gestel et al. showed that the decrease in the PICU mortality of children post-HSCT between 1994 and 2000 was no longer statistically significant after adjustment for the reduction in use of mechanical ventilation observed during this period [66]. Thus, it can be debated whether the overall improvement in mortality is secondary to the optimization of management and a less aggressive (or harmful) ventilatory strategy or is merely due to having less severely ill patients admitted to the PICU.

The prognosis of children post-HSCT admitted to the PICU has to be put into perspective with their overall prognosis, which has substantially improved. The rate of complications that may lead to a PICU admission, such as GVHD, sepsis, and respiratory failure, has decreased as well as the hospital mortality (from 12% in 1997 to 6% in 2003 (*p* < 0.001)) [65]. In a recent study, Gooley et al. [67] compared patients transplanted in 1993–1997 and in 2003–2007 and found a 41% decrease in mortality (*p* < 0.001) along with a significant reduction of almost all of the complications analyzed. Several factors have been identified as contributing to these improved outcomes: more judicious patient selection for transplantation, less toxic conditioning regimens, improved surveillance and early management of infectious complications, changes in the management of GVHD, new ventilatory strategies, and changes in management of severe sepsis [57,65,67].

Predictors of poor prognosis regularly reported in children post-HSCT include the use of mechanical ventilation [18,19,22,23,25,66] (especially in the presence of lung disease [18,25,63,66]), the use of inotropic support [21,25,60], multiorgan failure [7,18,19,63], and the need for renal replacement therapy [18,23,60]. Other prognostic factors, such as hyperbilirubinemia [18,64,68,69], GVHD [6,18,19,25,60], and the type of transplant [18,21,23,25], are more controversial. The underlying condition that prompted the HSCT does not appear to affect prognosis [23,25] nor does the time elapsed between transplant and PICU admission [18,21]. All of these data must be interpreted with caution, because they result from single-center retrospective studies, often of small sample size. In the meta-analysis by van Gestel et al. [66], a decrease in mortality over time was observed in the univariate analysis, whereas mechanical ventilation was associated with an increased risk of mortality. Only pulmonary disease remained significantly associated with mortality in the multiple meta-regression analysis [66].

Composite scoring systems can be used to predict or to describe the outcome of groups of patients admitted to the intensive care [70]. These scores can be used for quality assessment, economical assessment, monitoring measurement, and research purposes. Commonly used prognostic scores seem to be of limited value in population of children post-HSCT, because they tend to underestimate the mortality of these patients [18,23,61]. The Pediatric RISk of Mortality score (PRISM score) evaluates the mortality risk based on data collected during the first 24 hours in the PICU [71]. Some authors have proposed to adapt this score by adding important prognostic factors for children post-HSCT, thus creating the Oncological-PRISM score (O-PRISM) [60]. An O-PRISM score ≥10 would suggest an increased risk of mortality [21,23,72]. Such a score, which has not yet been validated, could be used to better analyze cohorts of children post-HSCT admitted to PICU and in the evaluation of new treatment strategies.

### **Children post-HSCT and sepsis**

Severe sepsis/septic shock is still a major cause of PICU admission for children post-HSCT, although its incidence has decreased over the years thanks to the development of anti-infective strategies, the elaboration of reduced-intensity regimens, and better monitoring of subclinical infections [65].

The prognosis of severe sepsis is worse in children post-HSCT than in nontransplanted children with cancer. Fiser et al. analyzed 446 separate PICU admissions of 359 children with cancer with a diagnosis of septic shock (defined as cardiovascular dysfunction requiring fluid boluses or inotropic support in the presence of fever and suspected or proven infection) [73]. PICU mortality was 30% after HSCT versus 12% for other cancer patients (*p* < 0001). The logistic regression analysis identified HSCT as one of the factors significantly associated with mortality in children with cancer, requiring both mechanical ventilation and inotropic support (odds ratio 2.9; 95% confidence interval 1.1–7.4), together with PRISM score, fungal sepsis, and the need for multiple inotropes. Six-month survival was 69% among non-HSCT children versus 39% for children post-HSCT (*p* < 0.01). Although these mortality rates are substantial, they are not as bleak as previously feared and should encourage the provision of intensive management of severe sepsis in children post-HSCT.

### **Renal replacement therapy in children post-HSCT**

Children who have undergone HSCT are at high risk of fluid overload due to voluntary intravenous hyperhydration, infusion of multiple antibiotics, veno-occlusive disease, and multiple transfusions of blood products. In addition, the conditioning regimen may be associated with renal toxicity and with some degree of systemic inflammatory response syndrome accompanied by a capillary leak syndrome [74].

Preventing fluid overload is important in children post-HSCT, because it has been identified as a risk factor for PICU admission in a retrospective study [8]. Moreover, fluid overload worsens the prognosis of patients with hypoxemic respiratory failure [75], a leading cause of PICU admission for children post-HSCT.

The usefulness of renal replacement therapy (RRT) has been questioned in children post-HSCT. Several small retrospective studies have found a mortality rate ranging from 75% to 100% in this population receiving RRT [19,23,25]. However, Flores et al. recently reported more encouraging results. The authors reviewed data of 51 children post-HSCT from an American prospective registry of children receiving continuous RRT (CRRT) [76]. They found a PICU mortality rate of 55%. Patients requiring ventilatory support had a lower survival rate than the nonventilated patients (35% vs. 71%, *p* < 0.05).

RRT could be beneficial through other mechanisms than optimization of fluid balance. DiCarlo et al. reported a series of ten children with cancer (including six HSCT patients) who had developed ARDS and who seemed to have benefited from early high-volume RRT (continuous hemodiafiltration with a flow of 50 ml/min/1.73 m^2^) initiated at or near the start of mechanical ventilation, regardless of renal function [77]. Only four of these children had renal dysfunction and fluid overload. Nine of ten patients were extubated and eight survived (follow-up until 18 months after discharge from the PICU), suggesting that early initiation of CRRT in this population could prevent inflammatory lung injuries in addition to fluid overload. This concept is supported by the finding of Rajasekaran et al. of an association between high C-reactive protein serum levels at the end of CRRT and the risk of mortality (33 courses of CRRT among 29 patients) [69].

CRRT also may favorably influence oxygenation of children post-HSCT with ALI. Elbahlawan et al. published recently a retrospective analysis of a pediatric HSCT cohort admitted to the PICU and receiving CRRT during a course of mechanical ventilation for ALI [74]. An improvement in oxygenation (PaO_2_/FiO_2_ ratio) was observed 24 hours and 48 hours after the beginning of CRRT. This effect is likely to correlate with negative fluid balance. However, only five children survived to PICU discharge (17%).

Thus, it seems that RRT may be useful in children post-HSCT, particularly in the presence of fluid overload. The concept of using high-dose RRT to promote favorable clearance of inflammatory mediators is attractive, especially because pro- and anti-inflammatory systems are probably dysregulated in these patients. Further studies are required before this indication alone could be retained in current practice.

### **Proposal of a decisional algorithm for critically ill children with cancer**

**Figure 1:**
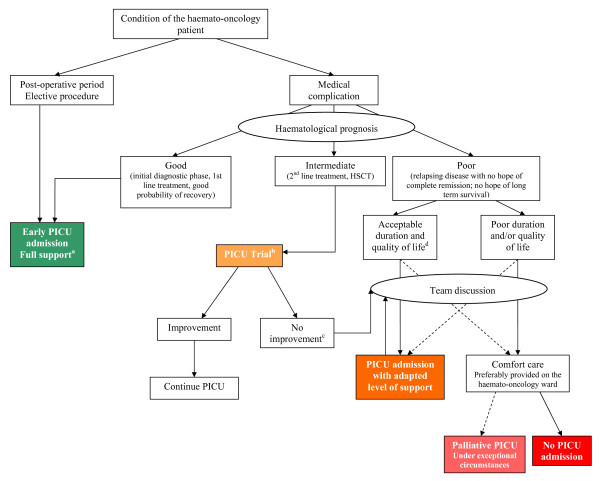
**Decisional algorithm for critically ill children with cancer.** PICU = pediatric intensive care unit; HSCT, hematopoietic stem cell transplantation; ECLS = extracorporeal life support. ^a^Unless a decision regarding limitation of care has been made before the intervention; ^b^The concept of PICU Trial is detailed in the text; ^c^New or progressive multiple organ dysfunction syndrome after days 3 to 5; ^d^May be defined as a Karnofsky score ≥ 50% and a life expectancy ≥ 100 days.

Discussions surrounding PICU admission of cancer children may raise difficult and sensitive questions, especially from an ethical standpoint. Establishing PICU admission criteria for children with cancer may facilitate these discussions. To this end, we propose an algorithm (Figure 1) adapted from the adult literature [78], based on consensus opinion of physicians caring for critically ill children with cancer at our institutions. As a result, some items remain subjective and are meant to be discussed case-by-case and modified as needed according to clinical settings. This highlights the paramount importance of constant dialogue between hemato-oncologists and intensivists, as well as with other health care professionals, the child, and his parents. This dialogue is essential to ensure continuity of high-quality care for these children with complex disease and evolving medical status and to benefit from the experience of all those involved in their care.

Similarly to the ICU admission policies recently proposed by Azoulay et al. [78], we offer different PICU admission strategies based on the anticipated prognosis of both the cancer and the condition leading to the PICU. Patients at low risk for mortality should be treated like the general PICU population. This category includes children admitted during the postoperative period and children in the initial phase of the disease. However, as mentioned previously, this classification is an aid to clinical decisions and should not be perceived as unchangeable. For example, some children may have an intermediate or poor prognosis even if they are in the initial phase of the disease, related to the severity of the cancer. The decision to offer a “full support admission” rather than a “PICU trial” (see below) has to result from a multidisciplinary discussion.

Like other authors [79], we consider that the concept of an “ICU trial,” developed and studied in the adult population [80], can be adapted to the pediatric population. The ICU trial consists of unlimited ICU support for a fixed time period, usually 3 to 5 days. The patient course is reviewed a few days after ICU admission, because their status at this point is thought to be more representative of their clinical condition and risk of mortality than if such estimation was based on first-ICU day values. This could help to identify patients who remain severely ill despite maximal therapy, with no improvement or with worsening condition, in whom difficult decisions, such as limitation of treatment, may be most appropriate [78,81]. In particular, the probability of survival is low in patients with persistent or worsening multiple organ failure after 48–72 hours [82-84], as well as in transplanted patients with mechanical ventilation exceeding 2 weeks [82]. As illustrated in the proposed algorithm, the ICU trial is particularly pertinent in critically ill patients with intermediate prognosis of their oncologic disease. With respect to patients with a poor hemato-oncologic prognosis (i.e., patients with no hope of survival), we believe that it is important to distinguish between two groups, according to the anticipated duration and quality of life. Mid-term life expectancy may be considered satisfactory in some patients, for example those having a Lansky score [85] (children aged 0–16 years) or a Karnofsky score [86] (children older than age 16 years) ≥ 50% and a life expectancy ≥ 100 days. The Lansky and the Karnofsky scores are validated performance status scales that are widely used to quantify the functional status of cancer patients [85,86]. They are frequently used as criteria for selecting patients for eligibility for phase I oncology trials. These patients could benefit from PICU support, after a discussion between the medical team and the family, to establish a reasonable treatment plan adapted to the patient’s needs. This plan usually excludes exceptional therapies (ECLS, RRT, high-frequency ventilation) and needs to be reassessed on a daily basis.

On the other hand, patients with an anticipated poor duration and/or quality of life will not benefit from PICU support. The concept of palliative PICU admission has been proposed [78] for patients in whom some specific form of comfort care cannot be provided on the ward; we believe that this situation is exceptional and should be avoided.

Future observational studies are warranted to evaluate this proposed algorithm for PICU admission in children with cancer.

## Conclusions

Children with cancer represent a population at risk for complications that may lead to PICU admission. Their prognosis has improved considerably during the past 20 years as a result of advances in hemato-oncology and intensive care. Children post-HSCT also have benefited from this progress, although they remain at a higher risk of mortality.

Most children with cancer should be admitted to the PICU if necessary and be considered eligible to receive maximal therapy. If after a few days of intensive treatment, there is absence of improvement, or progression of multiple organ failure, the care plan should be reviewed through adaptation of the level of support or guidance toward palliative care. Discussions pertaining to these important decisions should involve the intensivists, the hemato-oncologists, the family, and the patient when capable.

Large epidemiological studies in this population are scarce. Prospective, multicenter studies would lead to a better understanding of this population’s specificities and optimize the admission strategies as well as the management of these children in the PICU.

## Abbreviations

ALI, Acute Lung Injury; ARDS, Acute Respiratory Distress Syndrome; ECLS, Extra-Corporeal Life Support; GVHD, Graft Versus Host Disease; HSCT, Hematopoietic Stem Cell Transplantation; NIV, Non Invasive Ventilation; PICU, Pediatric Intensive Care Unit; RRT, Renal Replacement Therapy.

## Competing interests

The authors declare that they have no competing interests.

## Author’s contributions

PD carried out the literature review and analysis and drafted the manuscript. GE provided critical input into the draft manuscript. He particularly contributed to the section on respiratory failure. He had the original idea of a decisional algorithm, and drafted the first version with PD. GP, PH and PT all reviewed the draft and provided critical commentary. All authors read and approved the final manuscript.
